# Microsatellite isolation and marker development in carrot - genomic distribution, linkage mapping, genetic diversity analysis and marker transferability across Apiaceae

**DOI:** 10.1186/1471-2164-12-386

**Published:** 2011-08-01

**Authors:** Pablo F Cavagnaro, Sang-Min Chung, Sylvie Manin, Mehtap Yildiz, Aamir Ali, Maria S Alessandro, Massimo Iorizzo, Douglas A Senalik, Philipp W Simon

**Affiliations:** 1 Dept. of Horticulture, University of Wisconsin-Madison, 1575 Linden Drive, Madison, WI 53706, USA; 2 Estación Experimental Agropecuaria La Consulta, Instituto Nacional de Tecnología Agropecuaria, Ex Ruta 40. km 96, La Consulta CC 8, Mendoza (5567), Argentina; 3 Consejo Nacional de Investigaciones Científicas y Tecnológicas (CONICET), Argentina; 4 Dept. of Life Science, Dongguk University, 3-26 Pil-dong, Chung-gu, Seoul 100-715, South Korea; 5 Dept. of Horticulture, Faculty of Agriculture, Yuzuncu Yil University, 65080, Van, Turkey; 6 Department of Biological Sciences, University of Sargodha, Sargodha, Pakistan; 7 USDA-ARS, Vegetable Crops Unit, Dept. of Horticulture, University of Wisconsin-Madison,1575 Linden Drive, Madison, WI 53706, USA

## Abstract

**Background:**

The Apiaceae family includes several vegetable and spice crop species among which carrot is the most economically important member, with ~21 million tons produced yearly worldwide. Despite its importance, molecular resources in this species are relatively underdeveloped. The availability of informative, polymorphic, and robust PCR-based markers, such as microsatellites (or SSRs), will facilitate genetics and breeding of carrot and other Apiaceae, including integration of linkage maps, tagging of phenotypic traits and assisting positional gene cloning. Thus, with the purpose of isolating carrot microsatellites, two different strategies were used; a hybridization-based library enrichment for SSRs, and bioinformatic mining of SSRs in BAC-end sequence and EST sequence databases. This work reports on the development of 300 carrot SSR markers and their characterization at various levels.

**Results:**

Evaluation of microsatellites isolated from both DNA sources in subsets of 7 carrot F_2 _mapping populations revealed that SSRs from the hybridization-based method were longer, had more repeat units and were more polymorphic than SSRs isolated by sequence search. Overall, 196 SSRs (65.1%) were polymorphic in at least one mapping population, and the percentage of polymophic SSRs across F_2 _populations ranged from 17.8 to 24.7. Polymorphic markers in one family were evaluated in the entire F_2_, allowing the genetic mapping of 55 SSRs (38 codominant) onto the carrot reference map. The SSR loci were distributed throughout all 9 carrot linkage groups (LGs), with 2 to 9 SSRs/LG. In addition, SSR evaluations in carrot-related taxa indicated that a significant fraction of the carrot SSRs transfer successfully across Apiaceae, with heterologous amplification success rate decreasing with the target-species evolutionary distance from carrot. SSR diversity evaluated in a collection of 65 *D. carota *accessions revealed a high level of polymorphism for these selected loci, with an average of 19 alleles/locus and 0.84 expected heterozygosity.

**Conclusions:**

The addition of 55 SSRs to the carrot map, together with marker characterizations in six other mapping populations, will facilitate future comparative mapping studies and integration of carrot maps. The markers developed herein will be a valuable resource for assisting breeding, genetic, diversity, and genomic studies of carrot and other Apiaceae.

## Background

The Apiaceae family (order Apiales) contains a number of important vegetable and spice crop species including carrot, celery, fennel, cilantro, parsley and parsnip. Carrot (*Daucus carota *L.) is the most economically important species in the Apiaceae, with more than 21 million tons produced yearly worldwide (data for year 2003; http://faostat.fao.org/faostat). Carrot consumption has steadily increased in recent decades for several reasons, including a heightened awareness of its health-promoting attributes (carrots are the richest source of provitamin A carotenoids in the U.S. diet [1]), development of fresh-cut carrot products convenient for consumers, and adaptation of improved cultivars for warmer production areas [2].

Despite their economic importance, carrot and other Apiaceae have relatively underdeveloped molecular resources [3]. Three unsaturated linkage maps, mainly based on anonymous dominant AFLP markers, have been constructed in carrot [4,5] to assist breeding of this species. Inheritance studies on natural carotenoid mutants have identified factors conditioning root pigment accumulation [6], and both simply inherited pigment traits and QTL have been included in these maps [4,5,7]. The more recent addition of 22 genes from the carotenoid biosynthetic pathway [8], as well as Transposon-Display markers [9], onto one of the maps has increased both maker informativeness and coverage. The latter map, which was constructed using an F_2 _family derived from a cultivated × wild carrot cross, is considered the carrot reference map because it harbors important phenotypic traits, has fairly good coverage and includes the largest number of informative markers. However, direct comparisons between the reference and other carrot maps are currently difficult due to the lack of common markers across maps. This fact seriously limits the usefulness of these maps for assisting breeding, especially considering that a number of important carrot traits, including nematode resistance [10], anthocyanin pigmentation and reduced-sugar accumulation [4], have been mapped in unrelated genetic backgrounds. The lack of common markers across carrot maps is mainly due to an insufficient availability of informative and robust PCR-based markers. The carotenoid genes mapped by Just et al. [8] are not easily transferred to other maps with different genetic backgrounds, since they were mainly mapped by sequence detection of single polymorphism nucleotides (SNPs), rather than by fragment length polymorphisms, and because sequence conservation was very high (i.e., lack of polymorphism) in some carotenoid genes [8]. Thus, for these genes, little SNP polymorphism may be expected. The development of polymorphic and robust PCR-based markers in carrot, such as microsatellites, would facilitate their inclusion in different maps, thus serving as anchoring points for map integration. This would immediately increase map marker density, SSR-tagging relevant phenotypic traits and, perhaps, facilitate applications such as positional gene-cloning. In addition, other carrot genetic research studies, such as analysis of genetic diversity and phylogenetic reconstructions, previously approached using anonymous dominant AFLP markers [11] or laborious time-consuming codominant RFLPs [12], would also benefit from the development of microsatellite markers.

Microsatellites, or simple sequence repeats (SSRs), are the marker of choice in many molecular genetic applications including mapping, fingerprinting, genetic diversity, population structure analysis, gene flow and germplasm conservation studies. Their widespread adoption is due to several desirable characteristics: they are codominant, frequently and evenly distributed throughout genomes, selectively neutral, highly reproducible and rely on simple polymerase chain reaction (PCR) technology. In addition they are ubiquitous (SSRs can be found in nuclear and mitochondrial genomes of all organisms, as well as in plastid genomes) and hypervariable. The latter property is attributed to a high mutation rate of these repeats resulting from DNA polymerase slippage during DNA synthesis [13]. This mutational mechanism generates gains or losses of one or a few repeat units in the microsatellite, which accumulate more rapidly than point mutations and InDels [14], leading to a high number of alleles per locus. In plants, the high polymorphism found in microsatellites has allowed the detection of variability in species otherwise characterized by low levels of genetic diversity [15].

In Apiaceae, very few publicly available SSRs have been reported previously, and these were developed from carrot (9 SSRs [16]) and celery (11 SSRs [17]), the two most economically important species in the family. The availability of a large set of SSRs in carrot is likely to benefit research in other Apiaceae, since significant marker transferability has been observed across related taxa [18]. This is of particular interest to research groups working in minor crops or species with limited research funds; many laboratories have sufficient resources and expertise for running SSR-based PCR analyses, although perhaps not for the isolation and characterization of new loci.

In this study we report on the development of 300 new carrot SSR markers. Further characterization of these loci includes analysis of SSR distributions in genomic and EST sequence, linkage mapping onto the carrot reference map, evaluation of their mapping potential in subsets of seven carrot F_2 _mapping populations, evaluation of their potential for assessing genetic diversity among *Daucus carota *accessions, and evaluation of SSR marker transferability across 24 Apiaceae taxa.

## Results

### Distribution of microsatellites in genomic and EST sequence

Substantial variation in SSR frequency and distribution between genomic and EST sequence of carrot was found (Table 1). Because frequency distributions for GSSRs mainly reflect the library enrichment procedure (i.e., GSSRs are not a random sample of SSRs from genomic DNA), conditioning the type, length and sequence motifs of the SSRs obtained, only SSRs from BAC end sequence (BSSRs) were used as representatives of genomic SSRs in comparisons with SSRs from EST sequence (ESSRs). Overall, SSRs occurred at a lower density in genomic DNA (BAC ends) (134.5 SSRs/Mbp) than in ESTs (214.8 SSRs/Mbp). Dinucleotide (~17%), trinucleotide (~42%), and tetranucleotide repeats (~24%) predominated in genomic DNA, whereas in ESTs trinucleotides were the predominant repeats accounting for 50% of the total SSRs. The absolute density for this repeat type in EST data (108 trimers/Mbp) was nearly twice its density found in genomic sequence (56 trimers/Mbp). The same was observed for dinucleotide and hexanucleotide repeats, which were more than twice as frequent in ESTs (54.1 dimers/Mbp and 10.2 hexamers/Mbp) compared to genomic DNA (22.4 dimers/Mbp and 4.0 hexamers/Mbp) (Table 1). Conversely, the density of pentanucleotides, heptanucleotides and octanucleotides was more than 2 fold higher in genomic sequence than in EST sequence.

**Table 1:** Distribution of microsatellites GSSRs (BSSRs), and ESSRs) of carrot*

	GSSRs			BSSRs				ESSRs			
**SSR type**	**Count (%)**	**Mean # of repeat units/mean SSR length (bp)**	**Most frequent motifs**^‡^	**Count (%)**	**Mean # of repeat units/mean SSR length (bp)**	**Density **^ǂ ^**(SSR/Mb)**	**Most frequent motifs**^‡^	**Count (%)**	**Mean # of repeat units/mean SSR length (bp)**	**Density **^ǂ ^**(SSR/Mb)**	**Most frequent motifs**^‡^

Dinucleotide	116 (46.8)	10.0/21.2	AG(61), AC(34)	39 (16.7)	7.8/15.5	22.4	AT(46), AC, AG	207 (25.2)	8.1/16.5	54.1	AG(78), AC
Trinucleotide	28 (11.3)	7.0/21.3	AAC, AGT, AAG	97 (41.5)	4.3/12.9	55.7	AAG(27), AAC(17), AGT(13), ACT(12), AAG(27)	411 (50.1)	4.4/13.1	107.5	AAG(25), ACT(15), AGG(10), AGC(10)
Tetranucleotide	98 (39.5)	5.9/26.2	ACAT (59), AAAC	57 (24.4)	3.3/12.2	32.8	AAAT(33), AATT(21)	129 (15.7)	3.1/12.3	33.7	AAAG, AAAT
Pentanucleotide	2 (0.8)	3.0/15.0	-	27 (11.5)	3.0/15.2	15.5	AAAAG, AGCCG	29 (3.5)	3.1/15.7	7.6	AGCCC
Hexanucleotide	2 (0.8)	3.0/18.0	-	7 (3.0)	3.0/18.0	4.0	-	39 (4.8)	3.2/18.9	10.2	AAAAAG, AAAGAG
Heptanucleotide	0 (0)	3.0/21.0	-	4 (1.7)	3.0/21.0	2.3	-	4 (0.5)	3.0/21.0	1.0	-
Octanucleotide	2 (0.8)	3.0/24.0	-	3 (1.3)	3.0/24.0	1.7	-	2 (0.2)	3.5/28.0	0.5	-
Total perfect SSRs	248 (100)	7.9/23.1		234 (100)	4.4/13.9	134.5		821 (100)	5.0/14.2	214.8	
Compound SSRs	41 (16.5)	-/49.9		6 (2.6)	-/38.3	3.4		26 (3.1)	-/32.9	6.8	
Total seq. (Mbp)^ζ^	0.04			1.74				3.82			
GC content (%)	37.9			38.3				42.3			
											

* A minimum of 6 repeat units (r.u.) for dinucleotides, 4 r.u. for trinucleotides, and 3 r.u. for tetra-, penta-, hexa- hepta- and octanucleotides were used as parameters for searching microsatellites in genomic and EST sequence of carrot. ^ǂ ^Only density values for SSRs from BAC end sequences (BSSRs) and from ESTs (ESSRs) are presented (because GSSRs derive from an SSR-enriched library, analyses of this dataset would result in an overestimation of the SSR density in genomic DNA). ^‡ ^SSR motif considering complementary (e.g., "AAG" includes AAG + CTT motifs); SSR motifs occurring at a rate of at least 5% of the total are listed with occurrence as percentage within each SSR type class in parentheses; SSR classes without predominance of a particular motif are denoted as "-". ^ζ ^Total length of sequences analyzed. ^γ ^Includes both GSSRs and BSSRs.

Comparisons among microsatellites isolated from the different sequence datasets (GSSRs, BSSRs, and ESSRs) revealed that di-, tri-, and tetranucleotide repeats accounted for 82.6% to 97.6% of the SSRs among the three types evaluated, with di- and tetranucleotide motifs accounting for most of the GSSRs, while trinucleotide motifs were most common among BSSRs and ESSRs (Table 1).

Within genomic DNA, variation was also found between the two sequence datasets examined. Overall, GSSRs had more repeat units (7.9 *versus *4.4) and consequently were longer (23.1 bp *versus *13.9 bp) than BSSRs (*P *< 0.0001). The larger number of repeat units in GSSRs compared to BSSRs was evident and significant (*P *< 0.01) for di, tri, and tetranucleotides, whereas penta to octanucleotides had the same mean number of repeats in both datasets. SSR length had a similar relationship for these repeat types in both datasets. With regard to repeat types, GSSRs yielded a higher proportion of di and tetranucleotides, as compared to BSSRs, whereas trinucleotides and penta to octanucleotides were more frequent in the latter group. In addition, GSSRs included a significant fraction (16.5%) of often long, compound microsatellites, such repeats being nearly 7 times more frequent in this group compared to BSSRs.

Based upon posterior probabilities, the distributions of sequence motifs ((AT)_n_, (AC)_n_, (CG)_n_, etc.; (AAC)_n_, (ACC)_n_, (ACG)_n_, etc.) in the dinucleotide and trinucleotide classes were not random for BSSRs and ESSRs (Table 2). In BAC end sequence, (AT)_n _and (CG)_n _dinucleotides were more and less frequent than expected, respectively. In ESTs, (AT)_n _repeats were less frequent than expected whereas (AG)_n _and (CT)_n _dinucleotides occurred at a higher-than-expected incidence. AG and AC motifs occurred frequently in dinucleotide SSRs of all sequence origins, although AT dinucleotides were most frequent among BSSRs. Repeats of AAG, ACT, and AAAT were abundant and common to both genomic and ESSRs. On the other hand, repeats of AAC, AGT, ACAT, AATT, and AAAAG, predominated mainly among genomic microsatellites, whereas AGG, AGC, AAAG, AGCCC, AAAAAG, and AAAGAG motifs were most frequent in EST SSRs (Table 1). Comparisons between observed and expected trinucleotide repeat motifs presented no clear trends but observed distributions differed from those expected for many motifs to result in significant deviation based upon chi-square analysis (Table 2).

**Table 2:** Departure of dinucleotide and trinucleotide genomic (BAC end, BSSR) and EST (ESSR) motifs from expected distributions*

SSR type	χ^2^	df	p
BSSR dinucleotide	68	5	< 0.0001
BSSR trinucleotide	38	19	0.006
ESSR dinucleotide	6986	5	< 0.0001
ESSR trinucleotide	82	19	< 0.0001

*A minimum of 6 repeat units (r.u.) for dinucleotides were used as parameters for searching microsatellites in genomic and EST sequence of carrot. Chi-squared distributions indicate comparison of observed SSR distribution of SSR motif types ((AT)_n_, (AC)_n_, (CG)_n_, etc.; (AAC)_n_, (ACC)_n_, (ACG)_n_, etc.) to posterior probabilities. Tests are partitioned by library and sequence motif. χ^2 ^= chi-square statistic; df = degrees of freedom. SSRs were non-randomly distributed across motif types.

**Figure 1:**
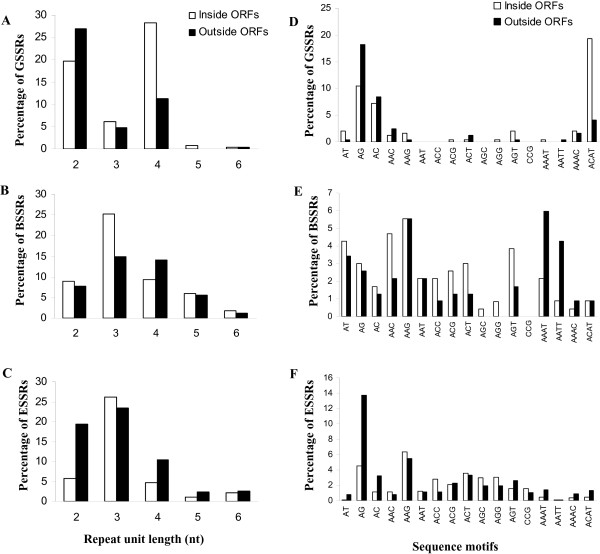
**Distribution of microsatellites in genomic and transcript sequences of carrot**. Frequency distribution (%) of predominant repeat types (A-C) and sequence motifs (D-F), by microsatellites origins GSSRs (A, D), BSSRs (B, E), and EST-derived SSRs (ESSRs) (C, F), inside and outside open reading frames (ORFs).

Microsatellite distribution was not uniform across coding and non-coding regions of carrot. Frequency distributions of both repeat types and sequence motifs for each microsatellite origin, i.e., a library enrichment procedure (GSSRs), BAC end-derived (BSSRs) and EST-derived SSRs (ESSRs), varied markedly across these DNA fractions (Figure 1). Among GSSRs, di- and tetranucleotide repeats were most common, and tetranucleotide repeats were distinctive in being the only GSSR repeat type with a significantly different occurrence inside and outside of ORFS, with over 70% of tetranucleotide SSRs inside ORFs. Analysis of repeats in BAC end sequence (BSSRs) revealed a predominance of trinucleotides in coding sequences, (accounting for 25% of the total SSRs and nearly half of the repeats found in ORFs) compared to non coding regions, whereas tetranucleotides were somewhat more abundant in non-coding regions. The overrepresentation of trinucleotides in ORFs of genomic DNA was higher than expected by chi-square analysis (Table 3) and associated to a high frequency of AAG, AAC, AGT, ACT, ACG, and ACC motifs, whereas non-coding regions rich in tetranucleotides were particularly GC-poor with an abundance of AAAT and AATT motifs (Figure 1). Dinucleotide, pentanucleotide, and hexanucleotide repeats were nearly equally frequent in protein coding and non-coding regions of BAC end sequence (Figure 1B).

**Table 3:** Placement and chi-square significance of microsatellite distribution inside and outside of ORFs from GSSRs, BSSRs, and ESSRs of carrot*

Repeat type	GSSRs		BSSRs		ESSRs	
	
	χ^2^	p (df = 1)	χ^2^	p (df = 1)	χ^2^	p (df = 1)
Dinucleotide	2.79	0.10	0.23	0.63	60.89	0.00
Trinucleotide	0.14	0.71	4.55	0.03	1.30	0.25
Tetranucleotide	18.00	0.00	2.96	0.09	17.06	0.00
Pentanucleotide	2.00	0.16	0.04	0.84	3.57	0.06
Hexanucleotide	0.00	1.00	0.14	0.71	0.42	0.52
Total	2.73	0.10	0.15	0.70	29.70	0.00

* A minimum of 6 repeat units (r.u.) for dinucleotides, 4 r.u. for trinucleotides, and 3 r.u. for tetra-, penta-, hexa-, hepta-,and octanucleotides were used as parameters for searching microsatellites in genomic and EST sequence of carrot. Chi-square distributions indicate observed SSR motif types inside versus outside ORFs as compared to posterior probabilities. χ^2 ^= chi-squared statistic; df = degrees of freedom. Observed distributions generally fit those expected (p ≥0.05) except for tetranucleotides for GSSRs (from a hybridization-based enrichment of genomic library), and trinucleotides for BSSRs (from BAC end sequence). For ESSRs (from ESTs) the observed distributions only fit expected ones for tri-, penta-, and hexanucleotide repeats, with observed occurrence of di- and tetranucleotide repeats occurring much more often outside ORFs than expected.

The distribution of SSR types in EST sequences was especially variable, with trinucleotides predominating in ORFs, representing more than 65% of the SSRs found inside ORFs (data not presented) and 26% of all ESSRs, and dinucleotides and tetranucleotides predominating in non-protein coding sequences of the ESTs (i.e., mainly UTRs) (Figure 1C). In EST ORFs, the most frequent trinucleotide motifs were AAG, ACT, AGG, AGC, and ACC. In the dinucleotides-rich UTR region of ESTs, AG and AC motifs were three times more frequent than in protein-coding regions of ESTs (Figure 1F).

### SSR marker development

Primer pairs for 156 GSSR and 144 BSSR loci were designed. Of these 300 primers pairs, 243 flanked single SSRs (202 perfect repeats and 41 compound SSRs) and 57 flanked multiple SSRs (i.e., the template sequence flanked by the primers annealing sites included more than one SSR). Markers with single perfect repeats included 59 dinucleotides, 56 trinucleotides, 64 tetranucleotides, 10 pentanucleotides, 7 hexanucleotides, 3 heptanucleotides, and 1 each of mono, octa and nonanucleotides. Further information on these 300 SSR markers is presented in Additional File 1 - Table S1, including primer sequence, annealing temperature, repeat motif and its position in template sequence, expected amplicon length, and the template DNA sequence carrying the SSR (for developing alternative primers if desired). For a number of microsatellites detected computationally, we were not able to design primers because they either lacked suitable flanking sequences or the total sequence length was too short (< 120 bp).

### Marker polymorphism analyses in carrot F_2 _families

A total of 300 SSRs (156 GSSRs and 144 BSSRs) were successfully characterized in subsets of 7 carrot F_2 _families (Table 4). Details on the performance of each SSR marker in the different mapping populations are presented in Additional File 1 - Table S2. Overall, 196 SSRs (65.1%) were polymorphic in at least one mapping population. These included 120 GSSRs and 76 BSSRs. Of particular interest for map merging is the fact that 123 SSRs (87 GSSRs and 36 BSSRs) were polymorphic in two or more mapping populations, suggesting that these common markers may serve as anchoring points across maps. Overall, the percentage of potentially mapable markers in the F_2 _families, as resolved by high-resolution agarose gel electrophoresis, ranged from 17.8% (in population #4, Table 4) to 24.7% (population #5). Codominant segregation was observed in 38% (population #2) to 78% (population #6) of the segregating markers, whereas dominant segregation accounted for 22% (population #6) to 62% (population #2) of the segregating markers.

**Table 4:** Carrot F_2 _mapping populations used to evaluate SSR markers

**Ref**.	Pedigree	Phenotypic segregating traits of interest	Reference
**1**.	B493 × QAL	QTL for carotenoid accumulation (22 structural carotenoid genes mapped)	Just et al. (2007) [7], Just et al. (2009) [8]
**2**.	HCM × Brasilia	QTL for carotenoid accumulation	Santos and Simon (2002) [5]
**3**.	B9304 × YC7262	*Y_2 _*- Differential xylem/phloem carotene levels *Rs *- Sugar type (reducing/non-reducing) in roots *P_1 _*- Purple/yellow pigment accumulation in roots	Vivek and Simon (1999) [4]
**4**.	Biennial5 × Criolla INTA	*Vern1 *- Vernalization requirement *Rf1 *- Fertility restoration	Unpublished
**5**.	70349 [(SNts × Camberly) × (Turkish × 2566B)]	*Y_2 _*- Differential xylem/phloem carotene levels *Rs *- Sugar type (reducing/non-reducing) in roots *P_1 _*- Purple/yellow pigment accumulation in roots *P_2 _*- Purple/green petiole	Unpublished
**6**.	10117 (PI173687 × B10138) × (PI173687 × B493)	*Y_2 _*- Differential xylem/phloem carotene levels *P_1 _*- Purple/yellow pigment accumulation in roots	Unpublished
**7**.	(Ping Ding × YC7262) × 8542	*Mj_2 _*- Resistance to *Meloidogyne javanica*	Unpublished

Substantial variation in the degree of polymorphism was found between the two sets of markers (GSSRs and BSSRs). In general, GSSRs were more polymorphic than BSSRs. Depending on the F_2 _family, 21.7 - 35% (mean 29.6%) of the GSSRs, and 6.3 - 17.4% (mean 12.8%) of the BSSRs, were polymorphic. Overall, nearly 77% of GSSRs and 52% of BSSRs were polymorphic in at least one F_2 _family. The mean polymorphism index (PI), which takes in account the type of polymorphism (e.g., dominant, codominant), was significantly higher (*P *< 0.001) for GSSRs (23.6%) compared to BSSRs (9.8%), regardless of the mapping population.

**Figure 2:**
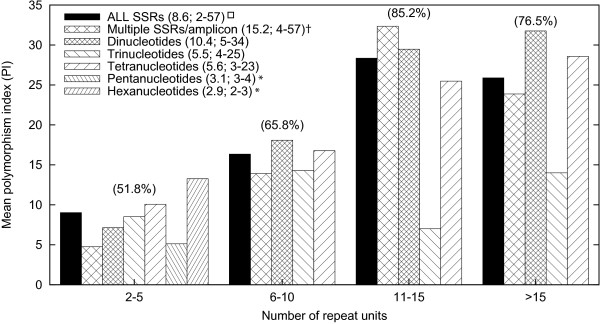
**Relationship between polymorphism and repeat number classes for perfect microsatellites**. Polymorphism Index values were calculated based on marker evaluations in 7 carrot F_2 _families as described in materials and methods. Numbers in parenthesis above the bars indicate the percentage of polymorphic markers for a particular repeat number class (i.e., the % of markers that were polymorphic in at least one F_2 _population). The mean number and range of repeat units for each SSR type are provided in the legend. *All penta and hexanucleotides repeats had less than 5 repeat units and, thus, were only included in the first class. ^† ^For SSR markers that included more than 1 perfect microsatellite in their amplicon sequence the sum of all repeat units was considered [e.g., (AT)6, (CTT)5 = 11 repeats].  ^☐^ Includes all markers with perfect SSRs.

SSR polymorphism -as evaluated in 7 mapping populations- was related to repeat number. Figure 2 presents mean polymorphism indexes for perfect microsatellites across four repeat number classes. A general trend showing increasing rates of polymorphism associated with increased repeat number was observed. Supporting this observation, significant correlations (*P *< 0.0001) between number of repeat units -and total SSR length- and polymorphism index (PI) were detected. Also, the percentage of polymorphic loci (i.e., the % of markers that were polymorphic in at least one F_2 _population) followed a similar trend across the repeat number classes, with values of 51.8%, 65.8%, 85.2% and 76.5% for SSR markers with < 6, 6-10, 11-15, and > 15 repeat units, respectively (Figure 2).

Markers harboring multiple perfect microsatellites in their amplicon sequence (i.e., "multiple SSRs/amplicon") with 11-15 repeat units were the most polymorphic markers, followed by long dinucleotides and tetranucleotides with more than 11 repeat units. With the exception of trinucleotides, for which there was no clear increase in polymorphism with increased repeat number, the SSR markers considered altogether revealed a clear positive relationship between the two variables, with a nearly 3-fold increase in polymorphism when comparing the lowest (< 6; PI = 9%) and highest (> 15; PI = 26%) repeat number classes.

### Marker transferability across Apiaceae

A total of 300 SSR loci were assessed across 23 Apiaceae accessions for a total of 6,900 primer/accession combinations. Of these, 4,346 (63%) produced fragments within the expected size range. Combinations that produced fragments outside the expected size range (i.e., > 100 bp larger or smaller than the original carrot sequence) were considered non-specific amplifications and regarded as negative results. This range was arbitrarily selected to simplify the analysis, especially in the cases where more than two bands were amplified. All successful amplifications were obtained at annealing temperatures between 0 and -2° of the recommended value. Alternative PCR protocols, such as touchdown, did not significantly improve amplification success.

**Figure 3:**
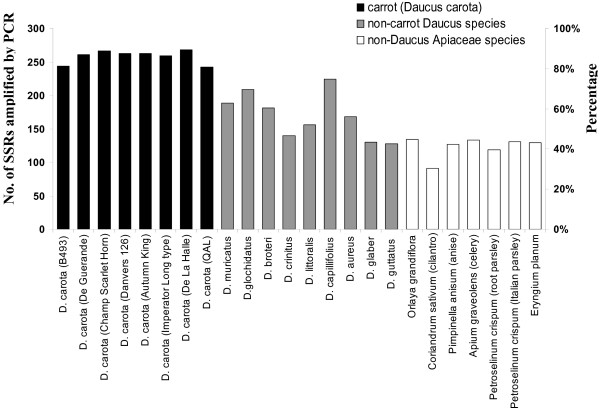
**Carrot SSR marker transferability across Apiaceae species**. Number and percentage of SSR markers that successfully generated PCR products of nearly expected size (i.e., the expected amplicons length for carrot, Additional File 1 - Table S1) in 23 Apiaceae accessions, including 8 accessions of carrot (1 inbred line, 6 commercial cultivars, and 1 wild carrot) (black bars), 8 non-carrot *Daucus *species (grey bars), and 7 non-*Daucus *Apiaceae species (white bars). Names of carrot cultivars and inbred lines, and the common names of non-carrot species are in parentheses.

The potential transferability of SSRs across Apiaceae taxa varied widely among the accessions (see Additional File 1 - Table S3 for details on the accessions used) and this was highly associated with the accessions' phylogenetic relatedness to carrot, the species from which the markers were developed (Figure 3). Thus, for carrot (*D. carota*) accessions, the total number of markers that produced amplicons of expected size was high, and ranged from 242 (81%), for the wild carrot 'QAL', to 268 (89%), in the Nantes-type French cultivar 'De La Halle', with a mean value of 258 (86%) markers. PCR amplification efficiencies in non-*carota Daucus *accessions were intermediate between carrot and non-*Daucus *Apiaceae accessions, with a mean of 175 (58%) successful amplifications, and ranging from 128 (43%) in *D. gutattus *to 224 (75%) in *D. capillifolius*. As expected, *D. capillifolius*, which is a close relative of *D. carota *and the only species in our data set with the same chromosome number as carrot (2*n *= 2*x *= 18), had the highest success rate, almost as high as the *D. carota *accessions (Figure 3). The more distantly related non-*Daucus *Apiaceae species generated SSR amplicons with 91 (30% in cilantro) to 134 (45% in *Orlaya grandiflora*) of the markers, with an average of 123 (41%) for this group. Again, the species most closely related to carrot within this group, *O. grandiflora*, had higher SSR amplification efficiency than other non-*Daucus *accessions.

Consistent with the previous analysis, the number of SSR primer pairs that produced expected-size amplicons across all the *Daucus carota *accessions (8), non-*carota Daucus *(16) and Apiaceae (23) was 200, 23, and 8, respectively. The performance of each SSR marker across the 23 Apiaceae accessions is presented in Additional File 1 - Table S4.

### Linkage mapping

**Figure 4:**
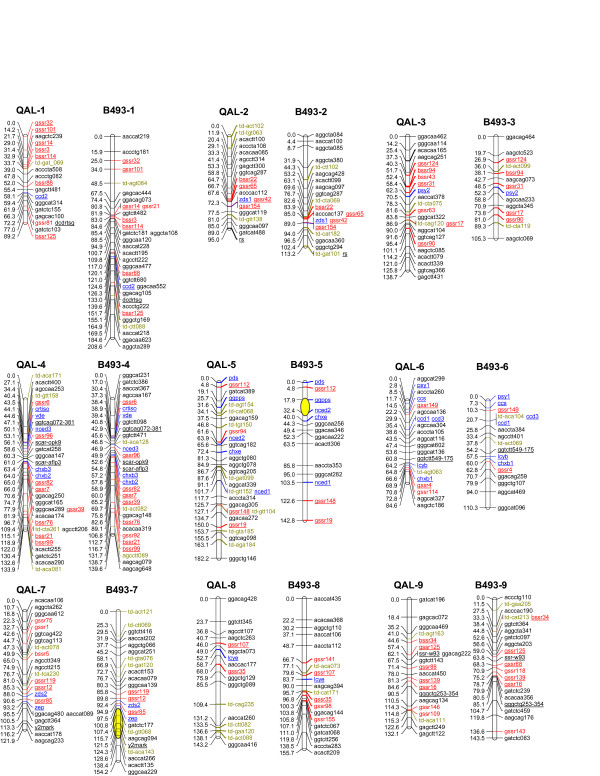
**Genetic linkage maps of the wild carrot parent QAL and cultivated parent B493**. Microsatellites mapped in this work are denoted in red. Carotenoid biosynthesis genes [8] and *Dc*MTD markers [9] are denoted in blue and green letters, respectively. Codominant markers present in both QAL and 493 maps are underlined. B493-map positions for significant QTL for root total carotenes (displaying 95% support intervals) in LG5 (former LG2) and LG7 (former LG5), were estimated based on data published previously [7,58], and represented by yellow circles. LGs were ordered and named according to their corresponding physical chromosomes [19].

Of the 300 SSR markers evaluated for polymorphism and mode of segregation in the B493 × QAL population (see Additional File 1 - Table S2), 170 (56.7%) were monomorphic and 66 (22%) polymorphic, whereas 28 SSRs (9.3%) did not produce amplicons and 36 (12%) yielded ambiguous band patterns that did not allow their inclusion in the previous classes. The polymorphic markers were assayed in the entire F_2 _population. Of these, 11 SSR markers were omitted because severe segregation distortion and/or multiple PCR products (presumably due to duplicated loci or non-specific amplifications) were observed. The remaining 55 markers -13 BSSRs and 42 GSSRs- generated robust and easily interpretable genotypes that could satisfactorily be used for individual genotyping and genetic mapping. These included 38 codominant and 17 dominant SSRs, which were successfully placed in the carrot reference linkage maps (Figure 4). No segregation distortion was detected in this SSR marker array as evaluated by F_2 _chi-square segregation analyses. The parental maps of QAL and B493 included 49 (39 codominant, 10 dominant) and 46 (39 codominant, 7 dominant) SSRs, respectively. These include a codominant SSR marker (ssr-w93) previously mapped in LG9. The mapped SSRs were distributed across all 9 linkage groups (LGs) of the carrot genome, with 2-8 and 2-9 markers/LG in the QAL and B493 maps, respectively. Only 5 and 3 map intervals with genetic distance greater than 20 cM (but smaller than 26 cM) scattered throughout different LGs were observed in the B493 and QAL maps, respectively, indicating a relatively evenly distributed map coverage. A comparative summary of both parental maps, by linkage groups, is presented in Table 5.

**Table 5:** Summary of the parental (QAL and B493) maps of carrot

		s	QAL map						B493 map					
**LG**	**Total # markers mapped**	**# markers common to QAL and B493 map**	**# markers mapped on the QAL map**	**# mapped markers segregating only from QAL**	**# SSR markers mapped**	Other mapped markers *	**LG size (cM)**	**Mean marker distance (cM) ^ǂ ^**	**# markers mapped on the B493 map**	**# mapped markers segregating only from B493**	**# SSR markers mapped**	Other mapped markers *	**LG size (cM)**	**Mean marker distance (cM) ^ǂ ^**

**1**	42	9	19	10	8	8A, 1T, 1G, 1S	89.2	5.0	32	23	8	20A, 2T, 1G, 1S	208.6	6.7
**2**	34	6	19	13	4	10A, 3T, 2G	95	5.3	21	15	4	11A, 4T, 2G	113.2	5.7
**3**	31	6	23	17	7	13A, 2T, 1G	138.7	6.3	14	8	5	6A, 2T, 1G	105.3	8.1
**4**	46	16	32	16	8	13A, 4T, 5G, 2S	133.9	4.3	30	14	9	11A, 3T, 5G, 2S	139.6	4.8
**5**	35	8	29	21	4	12A, 8T, 5G	182.2	6.5	14	6	3	6A, 5G	142.8	11.0
**6**	29	9	21	12	3	11A, 1T, 6G	84.6	4.2	17	8	2	7A, 2T, 6G	110.3	6.9
**7**	41	6	22	17	5	12A, 2T, 2G, 1S	121.9	5.5	23	18	2	12A, 6T, 2G, 1S	154.2	6.7
**8**	34	3	17	14	2	10A, 4T, 1G	143.2	9.0	20	17	5	12A, 2T, 1G	155.7	8.2
**9**	35	7	20	13	8	10A, 2T	132.1	7.0	22	15	8	12A, 2T	143.5	6.8
**Total**	327	70	202	133	49	99A, 27T, 23G, 4S	1120.8	5.9^†^	193	124	46	97A, 23T, 23G, 4S	1273.2	7.2^†^

* A = AFLPs; T = Transposon display markers; G = Gene-derived markers; S = SCAR markers

^ǂ ^Mean distance between markers is the arithmetic mean of map distances between adjacent markers shown in Figure 4.

^† ^Arithmetic mean of the mean distance between markers in each linkage group

Overall, after mapping the SSR loci, the linkage map of the wild carrot QAL contains 202 molecular markers (69 codominant and 133 dominant) covering 1,120.8 cM, with an average distance between adjacent markers of 5.8 cM, whereas the cultivated B493 map harbors 193 markers (69 codominant and 124 dominant) covering 1273.2 cM, with a 6.9 cM average marker distance. Thus, although the parental B493 map includes fewer markers, it has a larger total map length than the QAL map. A paired t-test revealed a significantly higher (*P *= 0.047) mean recombination fraction between adjacent markers (6.9 for B493 and 5.8 for QAL) when comparing the two parental maps. Although marginally significant, the higher mean recombination found in B493 may help explain its larger observed total map length.

Because in a very recent study [19] the linkage groups (LGs) from this map were integrated with actual chromosomes by means of flourescent in situ hybridization (FISH) mapping of BAC clones anchored by LG-specific markers (including some SSRs), the LGs in Figure 4 were named, ordered and oriented north-south according to the corresponding chromosomes. By convention, chromosomes are numbered consecutively from longest to shortest, and they are oriented with their short and long arms following north-south directions. Thus, correspondences between our LG designations and those from previous maps [5,8,9] are as follows: LGs 1, 3, 5, 6, and 7 correspond to former LGs 1, 8, 2, 3, and 5, respectively, with conserved orientation, whereas LGs 2, 4, 8, and 9, in this study (Figure 4) correspond to former LGs 4, 6, 9, and 7, respectively, with inverted north-south orientations.

The 38 fully informative (i.e., codominant) SSR markers consistently mapped to homologous LG pairs of the cultivated (B493) and wild (QAL) carrot genomes (i.e., all SSRs that mapped on the same LG in one parent also did so in the other parent). In addition, for these common markers, complete conservation of locus order was observed along the individual LGs between the two maps (Figure 4). This was also true for other -previously mapped- codominant markers (e.g., carotenoid genes, SCARs). Thus, no evidence of rearrangement of chromosomal blocks between the wild and the domesticated carrot subspecies was found.

Within each LG, SSRs were frequently positionally associated to genes. More than 40% of the SSR markers of both maps (19 and 18 SSRs in the QAL and B493 maps, respectively) mapped within 10 cM from previously mapped genes, whereas 16 of the 22 (~73%) carotenoid genes - in both, QAL and B493 maps- had 1 or more SSRs within a 10 cM distance, suggesting that these repeats are relatively frequent in genic regions of the carrot genome.

### SSR diversity in Daucus carota accessions

Our diversity analysis including 65 cultivated and wild carrots revealed valuable information on the degree of polymorphism of ten selected microsatellite loci. Table 6 presents the number of alleles (NA), allele lengths, and expected heterozygosity (*H_e_*) found for these SSRs in our *D. carota *diversity collection. For this germplasm, 190 different alleles, with lengths ranging from 144 to 433 bp, were identified. All the loci examined were highly diverse. The average number of alleles per SSR was 19.1 with a range of 10-29, whereas the mean expected heterozygosity was 0.84, and ranged from 0.77 for gssr9 to 0.91 for gssr4. The most polymorphic loci were gssr4 (NA = 29; *H_e _*= 0.91) and gssr6 (NA = 19; *H_e _*= 0.89), and the least polymorphic was gssr65 (NA = 10; *H_e _*= 0.79).

**Table 6:** General diversity statistics for 10 SSR loci evaluated in 65 *Daucus carota *accessions

Locus	Number of alleles	Allele size range (bp)	*H_e_*
gssr3	20	285-335	0.86
gssr4	29	253-320	0.91
gssr6	19	283-331	0.89
gssr9	22	281-337	0.77
gssr16	16	229-346	0.82
gssr35	21	144-219	0.87
gssr65	10	404-433	0.79
gssr85	14	219-294	0.84
gssr111	21	284-390	0.79
gssr134	18	272-334	0.8
total	190	144-433	
mean	19.1		0.84
s. d.	5.2		0.05

## Discussion

### Frequency and distribution of SSRs in carrot genomic and EST sequence

Microsatellite density in genomic DNA of carrot was estimated by analysis of 1.74 Mbp of BAC end sequence (the GSSRs dataset was excluded from this analysis because it derived from an SSR-enriched library and, therefore, its analysis would result in an overestimation of the SSR density in genomic sequence). Carrot had a rather low SSR density (134.5 SSRs/Mbp) compared to other species. SSRs analyses -using the same search parameters and program as with carrot- in the complete genome sequences of four model species revealed SSR densities of 370, 507, 529, and 508 SSRs/Mbp in *Arabidopsis thaliana*, grapevine, rice, and poplar, respectively. The lower SSR density in carrot compared to these species cannot be attributed to differences in the source of genomic sequence (BAC ends *versus *whole genomes) since analyses of BAC end sequence (BES) datasets from these and other species were also much more dense in microsatellites than carrot BES (data not presented). Similarly, transcript sequences of carrot (214.8 SSRs/Mbp), although more dense in SSRs than their genomic counterparts, were also less frequent in these repeats compared to ESTs of *Arabidopsis *(358 SSRs/Mbp), grapevine (247 SSRs/Mbp), poplar (425 SSRs/Mbp), soybean (403 SSRs/Mbp), rice (739 SSRs/Mbp), and sorghum (646 SSRs/Mbp).

Carrot trinucleotides were more frequent in transcripts than in genomic DNA. In addition, within BSSRs trinucleotide repeats occurred preferentially inside ORFs, and accounted for ~ 50% of the total SSRs found in these protein coding regions. The abundance of these repeats in ESTs and in ORFs is consistent with the notion that protein-coding sequences tolerate better frame-shift mutations (InDels) of 3 bp -or multiples of 3 bp- than other InDel lengths. Thus, trinucleotide repeats within coding sequences may translate fully functional proteins with a few extra (or fewer) aminoacids, whereas InDels of other lengths would translate abnormal, often deleterious, proteins. Consistent with our results, an overrepresentation of trinucleotides in protein-coding sequences has been reported previously in numerous plant species [20-24], as well as in other eukaryotes including humans, primates, rodents and insects [25,26]. The relative abundance of trinucleotides over other SSR types has been attributed not only to negative selection against frame-shift mutations in the coding regions but also to positive selection for specific single amino-acid stretches [21].

DNA polymerase slippage is the main mutational mechanism leading to changes in microsatellite length [13]. These changes in SSR size are most often gradual and step-wise since polymerase slippage only generates gains or losses of one or a few repeat unit(s) [27]. Thus, the fact that SSRs in carrot transcripts generally had fewer repeat units than SSRs in genomic sequence, even for trinucleotide repeats (trinucleotides were twice as frequent in ESTs compared to genomic data), suggests a negative selection pressure against microsatellite size increase in protein-coding sequences.

The non-random distributions of motif sequences among dinucleotide and trinucleotide SSRs of carrot included a higher than expected incidence of (AT)_n _repeats in genomic DNA (BAC ends), like that of several plant species including soybean, *Arabidopsis *and rice [22], but unlike the (AC)_n _predominant motif among dinucleotides in humans [25]. In contrast, the (AT)_n _motif was less often observed in ESTs than expected, while (AG)_n _and (CT)_n _were more common than expected. This may suggest different constraints for repeat motifs across diverse organisms.

### Marker development and analyses in F_2 _families

In this study, two different strategies were used for isolating and developing carrot SSR markers. The hybridization-based approach, as described by Glenn and Schable [28], yielded microsatellites (GSSRs) that were, in average, significantly longer (23.1 bp *versus *13.9 bp) and had more repeat units (7.9 *versus *4.4) than SSRs from BAC end sequences (BSSRs). These differences are, most likely, due to differences in the two strategies used. DNA library enrichment methods based on hybridization capture [28-31] are generally designed to yield a higher proportion of SSRs with large number of repeat units, targeting mainly long perfect repeats. Under this system, long DNA stretches of perfect repeats are hybridized more efficiently to the microsatellite probes and they are retained at a higher rate, compared to short repeats, during the washing steps, thus, increasing the relative proportion of long microsatellite sequences in cloned colonies [28]. Conversely, the BSSRs set represents a random sample -without enrichment for length, repeat type or sequence motif- from genomic DNA. Because of this, they provide a more reliable picture of the microsatellite distribution in the carrot genome. Longer and more repetitive SSRs have been obtained through hybridization-based methods compared to sequence-searches in other plant species, regardless of the type of DNA examined (i.e., genomic or ESTs), including *Brassica *[32,33], cotton [34], wheat and rice [24].

The differences in repeat number and length between GSSRs and BSSRs have important implications for marker potentiality, particularly with regard to polymorphism. In general, GSSRs were significantly more polymorphic than BSSRs, considering both the polymorphism index (PI) (23.6% *versus *9.8%) and the percentage of polymorphic markers (77% *versus *52%), and these differences were associated to a higher repeat number and length in the GSSRs group (as suggested by the significant positive correlations obtained between both variables and PI). Developments of SSR markers from other plant species, including cotton [35], barley [36] and pine [37], have also noted positive relationships between SSR polymorphism and number of repeat units. Together, these results are consistent with studies reporting that both SSR polymorphism and SSR mutation rate have a positive relationship with repeat number [38-40]. Concordantly, positive and significant relationships have also been found between repeat length and mutation rate in human [38], fruit fly [41] and yeast [42] microsatellites. These studies indicate that polymerase slippage, the main mutational mechanism in microsatellites [13], increases with higher repeat number and length, leading to a higher diversity in longer, more repetitive SSRs, as observed in the present study. However, contrary to these and our results, studies using markers developed from other plants, such as *Brassica *[32] and pearl millet [43], have reported lack of correlation between size of the SSR, both measured by length (bp) and repeat number, and detection of polymorphic loci. As pointed out in the latter two studies, SSR evolutionary age is a key factor for SSR diversity (i.e., recently evolved microsatellites would have fewer polymorphisms because of fewer occasions for mutation, even if they are relatively long) and this may help explain the lack of association found by them. In addition, most of the above studies (including ours) cannot rule out the possibility that InDels at regions other than the SSR motifs may account for some of the polymorphisms, thus influencing the expected relationship between length and polymorphism.

A major interest for evaluating the SSR markers in the carrot F_2 _populations was to assess their potential for mapping. Linkage maps using some of these F_2_s have already been constructed (see Table 4) and others are underway (Simon, personal communication). These maps include different phenotypic traits of interest (Table 4) and -before this study- they were mainly constructed using anonymous dominant markers, such as AFLPs and RAPDs, with only very few markers, or none, in common, thus, making their comparative analyses and/or integration difficult. The present work identified 123 SSRs (87 GSSRs and 36 BSSRs) that were polymorphic in two or more mapping populations, suggesting that these common markers may serve as anchoring points for merging carrot maps. Besides the inclusion of 56 SSR markers onto the carrot reference map (see below), work is underway in our lab to include these polymorphic SSRs in other maps with different genetic backgrounds (see Table 4). The integration of carrot linkage maps would enhance their usefulness for assisting breeding of this species, by increasing marker saturation nearby genes of interest and thereby facilitating applications like positional gene cloning, among others.

From our evaluation in seven carrot F_2 _families, 196 SSR markers (65%) were polymorphic in at least one mapping population. Because the PCR amplicons were size-separated using high-resolution agarose gel electrophoresis, which can only resolve fragments with size differences of at least 3 bp, a fraction of the markers evaluated in some populations, generated ambiguous band patterns. Although they may have been polymorphic, the bands were too close together in the gel to unambiguously score, and were classified as monomorphic (i.e., only unambiguously polymorphic and scorable markers were classified as "dominant" or "codominant" in Additional File 1 - Table S2). Thus, if other fragment separation systems, with better resolution, are used, such as separation of fluorescently-labeled fragments through capillary electrophoresis, the number of polymorphic markers may be expanded in some populations, particularly in cases of dinucleotide SSR markers varying in a single repeat unit.

High PCR amplification efficiencies were found in the F_2 _families for both sets of markers, GSSRs (83%) and BSSRs (87%). Comparable amplification efficiencies have been found in other plant species with SSR markers developed using hybridizations-based methods (~ 90% [44]) and sequence-based searches (85% [32]).

### Transfer success of carrot SSRs across Apiaceae

The availability of SSR loci for economically important species has increased interest in primer transferability to related taxa, especially for species in which molecular resources are limited. In Apiaceae, only a few publicly available SSRs have been reported previously, and these were developed from carrot (9 SSRs [16]) and celery (11 SSRs [17]), the two most economically important crop species in the family. Results from this study indicate that a significant fraction of carrot SSRs transfer successfully across Apiaceae. Locus amplification success was detected in 91 to 224 markers across 15 non-carrot Apiaceae species, including economically important crops like parsley (131 SSRs), celery (133 SSRs) and cilantro (91 SSRs). Prospects of a broader utilization of these markers beyond carrot include their application in taxonomic, population, and conservation studies as well as for mapping and assisting breeding in crop species.

It is, however, important to bear in mind that when using SSR markers across distantly-related species the amplification of a PCR product does not necessarily imply locus conservation, since size homoplasy, i.e. convergence in size of non-homologous fragments, may occur. Considering the possibility of this source of confusion, verification of the PCR product identity by sequencing has been suggested previously, particularly when working across genera and if there is uncertainty regarding the size range of the amplicons obtained [45]. However, verification through sequencing may not be necessary if working within the same genus as the species from which the SSRs markers were developed [46]. Thus, the use of carrot SSR markers for studies in non-*Daucus *Apiaceae should include verification, by sequencing, of the homology to the carrot SSR product sequence (see Additional File 1 - Table S1).

Transfer of carrot SSRs across *Daucus *species (carrot accessions excluded) was, in general, less successful than SSR transfer rate at the subgenus level reported for other species, whereas transfer of carrot SSRs across-genera was relatively higher than found in other plants. According to a previous review of SSR cross-transferability in plants [18], the average transferability across species in the same genus was 76.4%, and across related genera was 35.2%. We found these values to be 58.3% across *Daucus *species and 41% across the Apiaceae. However, it should be noted that SSR transfer success varied greatly across the different reports for both within-same genus (4.7 - 100%) and across different genera (0 - 71.4%) [18]. The huge variation found across these studies likely reflects differences in phylogenetic distance (and thus, also in conservation of sequences at priming sites) between the source and target taxa within each family, as well as differences in the number of taxa and SSR loci analyzed, and in the type of sequences used for marker development. For example, EST-derived SSRs are more conserved and thus they transfer across genera more readily than genomic SSRs [46]), among other factors.

Our data (Figure 3) suggest generally a higher rate of success in amplifying carrot SSRs in plants more closely related to carrot. This should not be surprising since closer-related taxa have higher overall sequence homology which translates to more conserved SSR flanking regions and, therefore, easier transferability of primer pairs. Negative relationships between SSR transfer success and phylogenetic distance between source and target taxa have been widely observed in many plant families [18,46].

The potential usefulness of SSR markers for diversity and phylogenetic studies in Apiaceae will depend, to a great extent, on the possibility that markers successfully amplify across different species and on the ability of the marker to detect polymorphism among the taxa. To have a preliminary picture of how suitable the SSR markers developed in this work may be for these applications, we investigated interspecific SSR variation among non-carrot species by analysis of amplicons sizes in the agarose gel images. Thus, for each SSR, the total number of different alleles in the non-carrot species dataset was recorded (Additional File 1: Table S4). Only SSRs that successfully amplified products in at least 80% of the non-carrot species (i.e., SSRs that generated amplicons in at least 12 of the 15 non-carrot species used to assess marker transferability) were considered. Overall, our results revealed 88 SSRs that generated amplicons in most (> 80%) outside-carrot species. Of these, 40 SSRs (29%) produced 3-9 different alleles (with an average of 4.9 alleles/SSR) in the non-carrot group. It should be noted that our calculation of 4.9 alleles/SSR in these selected markers is conservative, due to the low resolution of agarose gels which do not allow discrimination of different alleles varying in one or a few repeats. These results suggest that a significant proportion of the SSR markers developed herein may be suitable for addressing taxonomic or phylogenetic questions within Apiaceae.

Further analysis of the 88 SSRs that produced amplicons in the majority of the non-carrot taxa revealed interesting differences between the two SSR datasets. Although more BSSRs than GSSRs (52 and 36 markers, respectively) amplified successfully in most non-carrot taxa, GSSRs were much more polymorphic than BSSRs at the interspecific level. For example, among GSSRs 28 markers produced 3 or more different alleles (with a range of 3-9 and mean of 5.5), whereas only 12 BSSRs generated 3 or more alleles/SSR (with a range of 3-6 and mean of 3.6). It is likely that the generally higher polymorphism of GSSRs compared to BSSRs at the inter-specific level, which is in agreement with our results for both sets of markers in the carrot F2s, may be also due to the higher number of repeat units present in GSSRs.

### SSR linkage mapping

Prior to this work, important advances were made in the construction of carrot genetic maps with a range of molecular marker systems. Although some RFLPs [4] and a few SCAR and gene-specific markers were mapped [8], the most extensive genetic mapping data in carrot has been generated mainly with dominant AFLP, RAPD and Transposon-display (TD) markers [4,5,8-10]. While RFLPs are useful for comparative mapping purposes, high throughput genotyping and probe handling are difficult. Similarly, the carotenoid genes mapped by Just et al. [8] are not as easily transferred to other mapping backgrounds since their analysis relied in most cases on SNPs, due to the lack of larger polymorphisms (e.g., InDels) in these genes that can be scored as easily as SSRs. On the other hand, AFLP, RAPD and TD markers, while providing a relatively large number of markers per assay and good genome coverage, have limited information content and are not of much use for comparative mapping purposes and for validating QTL across pedigrees [8-10]. The addition of 55 SSR markers to the carrot reference linkage map together with detailed characterization of this novel set of 300 SSRs in subsets of six other mapping populations should allow significant advances in carrot comparative mapping and map-integration. The fact that most of the mapped SSRs were codominant (38 SSRs) in the B493 × QAL-derived population, with 2-8 informative markers per linkage group, together with the identification of putative codominant SSRs in other mapping populations adds extra value to the data published here for pursuing these goals. The inclusion of SSRs in linkage maps with additional pedigrees is currently underway.

The parental B493 map has a slightly larger total map length than the QAL map. Although the higher mean recombination found in B493 may help explain its larger map length, other factors -e.g., related to the type of markers used- may also cause this effect. Different recombination frequencies can be obtained just by sampling of the different markers, as well as errors derived from calculations of genetic distances from dominant markers data.

In the current map, we have modified linkage group designations and orientations, in accordance to recent cytogenetic data concerning the integration of carrot LGs with actual chromosomes [19]. Following standard conventions, consecutive numbers were assigned to the LGs in decreasing order of chromosome length (i.e., LG1 corresponds to the longest chromosome), and four LGs were inverted in their north-south orientations to agree with the standard short arm/long arm presentation of their corresponding chromosomes. It must, however, be noted that although all the LGs could be unequivocally associated to chromosomes, and thus their number designations are correct and complete, unambiguous LG orientations could only be defined for six of the nine LGs. Thus, chromosomes 4, 6, and 9 in the current map, which correspond to former LGs 6, 3, and 7, respectively, could not be unequivocally oriented, because a single anchored BAC probe was used for LG-chromosome integrations. Thus, their orientations were not modified from previous map versions [5,8,9]. However, the possibility remains that future cytogenetic data (for example by FISH analysis with several BAC probes SSR-anchored to these LGs) may reveal different orientations for these LGs. These modifications based on recent cytogenetic data, and the addition of 55 new SSR markers, add value to the updated reference carrot linkage map presented herein. Overall, the current maps involve 193-202 mapped loci, including 69 highly informative markers which consist of SSR, carotenoid gene and SCAR markers, spanning 1,121-1,273 cM, making it the most comprehensive genetic linkage map in the Apiaceae to date.

The SSR loci mapped across all 9 LGs in both parental maps, and they were distributed fairly evenly within most individual LGs, thus recommending their usefulness as anchor points for merging carrot maps. In addition, such dispersed map distribution of the SSR loci, has allowed us to develop BAC FISH probes carrying SSR sequences mapped to specific LGs. These SSR-anchored probes were used for integrating some LGs of carrot with chromosomes by FISH mapping [19].

The positional association observed between SSRs and previously mapped genes suggests that these tandem repeats are frequent in genic regions of the genome. This is in agreement with results of Morgante et al. [21], demonstrating higher microsatellite frequencies in the transcribed and non-repetitive fractions of plant genomes. One SSR (gssr112) and two SSRs (gssr12 and gssr119) in LG7 and LG5, respectively, were located in the vicinity of two highly-significant quantitative trait loci (QTL) for total root carotene accumulation. These correspond to the *Y *and *Y_2 _*loci, respectively, described by Buishand and Gabelman [47]. Microsatellites gssr12 and gssr119, although not tightly linked to *Y_2_*, may be useful for marker assisted selection, either as a complement of, or as an alternative to the lack of amplification of other robust more-closely linked markers, such as *Y2mark *[48].

## Conclusions

This work reports on the development and characterization at various levels of a novel set of 300 carrot SSR markers. Analysis of the distribution of SSR motifs, repeat lengths and polymorphism across genomic and EST sequences, as well as concerning different SSR isolation methods (hybridization-based versus sequence SSR-mining) may help decide over future strategies for developing valuable SSR markers in this and other species. The genetic mapping of 55 SSR loci onto the reference carrot linkage map, distributed throughout all 9 linkage groups, together with the characterization of the entire set of markers in 6 other mapping populations, should facilitate comparative mapping studies and integration of carrot maps. Particularly important for these purposes are the 38 codominant SSRs added to the map, which resulted in more than doubling the original number of informative markers in this -or any other- carrot map reported to date. In addition, SSR evaluations in carrot-related taxa indicates that a significant fraction of the carrot SSRs transfer successfully across Apiaceae, with heterologous amplification success rate decreasing with the target-species evolutionary distance from carrot. Nonetheless, a fairly large number of potentially useful SSR markers were identified for non-carrot *Daucus *(128-224 SSRs) and non-*Daucus *Apiaceae (91-134 SSRs) species, increasing the prospects of their successful utilization in other Apiaceae. In addition, allelic diversity at selected SSR loci was evaluated using 65 *D. carota *accessions. In this germplasm, the microsatellites proved to be highly polymorphic, with an average of 19 alleles/locus and 0.84 expected heterozygosity. The marker resources developed in this work should be a valuable tool for carrot breeding and genetics.

## Methods

### SSR identification and marker development

Two different approaches were used to isolate carrot genomic SSRs: 1) Construction and sequence analysis of a carrot (inbred line B493) genomic DNA library enriched for SSR loci (GSSRs) and 2) Bioinformatic mining for SSR motifs in a 1.7 Mbp BAC-end sequence (BES) database (BSSRs). GSSRs were developed at the Savannah River Ecology Laboratory, University of Georgia, using a hybridization capture approach for genomic library enrichment, as described by Glenn and Schable [28]. The DNA clones were sequenced from both directions using standard Sanger cycle-sequencing, and SSRs were detected using the program MISA [49]. The same software was used for the identification of BSSRs in 2,696 carrot BES (NCBI acc. # FJ147695-FJ150390) derived from inbred line B8503 [50]. Only SSRs with repeat length ≥12 nt and 3 or more repeat units were considered. Primer pairs flanking 156 GSSRs and 144 BSSRs were designed with *Primer 3 *(v.0.4.0).

For comparison purposes only (no markers were developed) a 3.82 Mbp EST dataset generated from 10-week old carrot (inbred B493) roots, was mined for microsatellites using the same programs and parameters described above. The EST dataset comprised 7,285 unique transcripts, 4,044 contigs and 3,241 singlets, which derived from initial analyses and processing (i.e., cleaning, trimming and assembly) of 18,044 Sanger sequence reads. SSRs in the EST dataset with a repeat length ≥12 nt and 3 or more repeat units were included. The resulting data were compared with microsatellites found in genomic DNA sequence (i.e., GSSRs, BSSRs).

For comparisons with carrot, the complete genome sequence of *Arabidopsis thaliana *L. (119.2 Mbp; accs. NC003070-71, NC003074-76), rice (*Oryza sativa *L.; 370.8 Mbp; accs. NC008394-405), grapevine (*Vitis vinifera *L.; 303.1 Mbp; accs. NC012007-25), and poplar (*Populus trichocarpa *L.; 307 Mbp; accs. NC008467-85) were downloaded from the National Center for Biotechnology (NCBI) database (Genomes Section), and mined for SSRs using the same search parameters and software. As a source of transcript sequences we used plant gene indices of The Institute of Genome Research (TIGR), which are non-redundant (unigenes) EST collections [51]. Thus, gene indices of *Arabidopsis *(74.8 Mbp; AGI.release_13), poplar (67.6 Mbp; PPLGI.release_4), grapevine (81.4 Mbp; VVGI.release_6), *Medicago truncatula *(51.9 Mbp; MTGI.release_9), soybean (51.3 Mbp; GMGI.release_13), rice (158.2 Mbp; OGI.release_17) and sorghum (32.4 Mbp; SBGI.release_9) were downloaded from the Gene Index databases (http://compbio.dfci.harvard.edu/tgi/) and searched for SSRs.

A custom Perl program performed a computational ORF detection, which was a search in each possible reading frame for an ATG start codon followed by a stop codon (TAA, TAG, or TGA) at a distance of 100 nt or greater, with no intervening start or stop codons in that reading frame. If two ORFs in different reading frames overlapped, the longer ORF was selected and the shorter ORF was disregarded. Details on the program used for finding ORFs are included in Additional File 2. SSRs, detected by MISA, were categorized as being either inside or outside ORFs. SSRs bridging an in-frame to out-of-frame boundary were discarded from further analysis.

#### Statistical analyses

The *t*-test statistic was used to compare SSR frequencies among the datasets using the program STATGRAPHICS Centurion XV. Evaluation of differences in repeat number across the GSSR, BSSR, and ESSR datasets included chi-square goodness of fit tests to compare observed SSR distributions within each dataset with regard to 1) distribution across sequence motif, 2) distribution of repeat motif and 3) distribution of SSR as inside or outside identified ORFs, using only those clearly categorized. Posterior probability distributions for GSSRs, BSSRs, and ESSRs were calculated separately. Sequence motif distribution posterior probability was calculated from the overall base composition of each dataset. Posterior probability distributions for SSR placement inside or outside ORF regions was based on the ORF sequence distribution within each dataset.

### PCR conditions and electrophoresis

PCR reactions were performed in 15 μl volume containing 7.15 μl water, 1.5 μl 10 × DNA polymerase buffer, 1.2 μl dNTPs (2.5 mM each), 1 μl of each primer at 5 μM, 0.15 μl *Taq *Polymerase at 10 u/μl (Promega, Madison, Wisconsin, USA) and 3 μl of genomic DNA. Thermocyclers were programmed as follows: initial denaturation at 94°C for 3 min., followed by 40 cycles of 94°C for 20 sec., appropriate annealing temperature for 1.0 min., and 72°C for 1.0 min., and a final step at 72°C for 5.0 min. Electrophoresis was carried out for 4-5 hours at 200 V on 4.5% high-resolution agarose (GenePure Hi-Res Agarose, ISC Bioexpress, Kaysville, UT) TAE gels supplemented with 4 ul (5.0 mg/ml) of ethidium bromide for each 100 ml of TAE.

Different methods for marker generation and analyses, including primer labeling, PCR conditions and separation of amplicons, were used for the genetic diversity analyses (described in detail in section "diversity analysis").

### Marker analyses in carrot F_2 _families

Since all carrot linkage maps reported to date were constructed using predominantly anonymous dominant markers such as AFLPs and this has severely limited map merging [52] microsatellite markers were developed to serve as anchor points across carrot maps. All SSR primer pairs (156 GSSRs, 144 BSSRs) were evaluated in samples (16 DNAs) from 7 carrot F_2 _mapping populations, as well as in the parental DNAs (when available). Information regarding the populations is presented in Table 4. Markers were evaluated based on their PCR amplification efficiency (i.e., whether an amplicon of expected size was generated) and polymorphism. For the latter, a polymorphism index (PI) was developed according to the formula: PI = [(2C + D)/(7 - nd) × 2] × 100, where 'C' is the number of populations for which the markers was codominant, 'D' is the number of populations for which the marker was dominant, 'nd' populations without information on the performance of the marker.

Simple regression analyses were performed between PI and other characteristics of the SSR markers (e.g., number of repeat units, repeat length, etc.) to investigate possible microsatellite features associated with polymorphism. For this purpose, the program STATGRAPHICS Centurion XV was used.

### Marker transferability across Apiaceae

To evaluate the potential utilization of SSRs within *Daucus carota *as well as in other carrot-related taxa, the GSSRs and BSSRs were tested in a sample of 23 Apiaceae accessions including 8 accessions of carrot (*D. carota*) (1 inbred line, 1 wild carrot, and 6 commercial cultivars), 8 accessions of non-carrot *Daucus *species, and 7 accessions of non-*Daucus *Apiaceae species. Accessions that produced an amplicon of the approximate length expected for that particular SSR in carrot were considered as successful PCR amplifications. PCR products were generated and resolved as described previously.

### Linkage mapping

#### Plant materials and DNA extraction

A genetic linkage map was constructed using a subset of 103 individuals from a previous F_2 _mapping population derived from the cross between the white-root wild carrot Queen Annes Lace (QAL) and the cultivated orange-root carrot inbred line B493. Details concerning the development of the mapping population, plant cultivation, DNA extraction, and detection/scoring of previously mapped AFLP, SCAR, *DcM*TD (*DcMaster *Transposon Display), and gene specific markers were described before [5,8,9,52].

#### Generation and analysis of marker data

PCR reactions were performed in the same way as the SSR reactions described above.

For purpose of marker genotyping, the F_2 _DNAs were analyzed in parallel with controls DNAs of QAL and B493. The genotypes of polymorphic SSRs were recorded as follows: homozygous maternal (B493) "A", homozygous paternal (QAL) "B", heterozygous "H", not A "C", not B "D", and missing data "-". The degree of segregation distortion associated with newly identified SSRs was determined by marker data comparison against the expected ratio of 1:2:1 (A:H:B) for codominant, and 3:1 (C:A or D:B) for dominant markers for an F_2 _using chi-square tests, where significant distortion was declared at *P *< 0.01 [53]. Separate maps were constructed for each parent to avoid problems related to the use of repulsion phase dominant markers, as described previously [52]. Dominant markers from a single parent linked in coupling were used in conjunction with all codominant markers. Linkage maps were constructed with MapMaker/EXP 3.0 [54], where markers were associated with the 'group' command at LOD = 4.0 and a maximum recombination frequency of 0.30. Markers within a group were ordered using 'three point' analysis followed by the 'order' command. Remaining markers were located using the 'try' command, and the map order was re-tested using the 'ripple' command. Recombination frequencies were converted to centimorgans (cM) using the Kosambi function.

### SSR genetic diversity in Daucus carota

#### Plant materials and DNA extraction

A total of 65 *Daucus carota *accessions were used in this study, including 50 cultivated carrots (*D.c*. spp. *sativa*) and 15 wild relatives of carrot (12 accs. of *D.c*. spp. *carota*, and one accession each of *D.c*. spp. *azoricus*, *D.c*. spp. *hispanicus*, *D.c*. spp. *drepanensis*) (Additional File 1 - Table S5). The cultivated carrot accessions included 8 inbred lines and 42 open-pollinated cultivars with representatives from 14 of the 16 European primary cultivars [6], and the predominant cultivars from North America ('Imperator') and Asia ('Kuroda'). For evaluation of morphological traits the carrot accessions were grown in commercial carrot fields in Wisconsin and California. For DNA extractions, plants were grown in greenhouses in Madison, Wisconsin, and genomic DNA was extracted from single plants as described by Murray and Thompson [55].

#### PCR amplification and fragment analysis

Ten unlinked microsatellite loci (gssr3, gssr4, gssr6, gssr9, gssr16, gssr35, gssr65, gssr107, gssr85, and gssr111) were used to investigate genetic diversity among cultivated and wild *Daucus *accessions. Each amplification reaction was performed in a 10 μl final volume, and included ~ 50 ng of DNA template, 0.65 U of Taq DNA polymerase (EconoTaq™), 1 × PCR buffer with 1.5 mM MgCl_2 _(EconoTaq™), 0.15 μM of reverse primer, 0.15 μM of M13-tailed forward primer (a universal M13 tail, labelled either with 6-FAM, HEX or NED fluorochromes, was added to the 5'-end of the forward primer to enable fluorescent labelling of the amplicons as described by Shuelke [56]), 100 μM each of the deoxynucleotidetriphosphates (dNTPs), 0.1 mg/ml bovine serum albumin (BSA), and 1% (w/v) of polyvinylpyrrolidone (PVP). The amplification conditions were 94°C for 4 min; 40 cycles of 94°C for 20 sec, 55-60°C for 1 min, 72°C for 1 min; and a final extension step of 72°C for 5 min. Estimation of amplicon lengths and microsatellite genotyping was performed at the University of Wisconsin Biotechnology Center using an ABI 3730*xl *capillary sequencer and GeneMarker software version 1.5 (SoftGenetics, State College, Pennsylvania).

#### Data analysis

General statistics, such as number of alleles (NA), allele length, and expected heterozygosity (*H_e_*), were calculated for these markers using the software Arlequin ver 3.1 [57].

## Abbreviations

AFLP: amplified fragment length polymorphism; RFLP: restriction fragment length polymorphism; EST: expressed sequence tag; Mbp: million base pairs; PCR: polymerase chain reaction; SNP: single nucleotide polymorphism; RAPD: random amplified polymorphic DNA; SSR: simple sequence repeats; QTL: quantitative trait loci; LG: linkage group; PI: polymorphism index; FISH: fluorescent in situ hybridization; BAC: bacterial artificial chromosomes; BES: BAC-end sequence; SCAR: sequence characterized amplified regions; BSSRs: SSRs from BAC end sequence; ESSRs: SSRs from EST libraries; GSSR: SSRs from genomic library enrichement.

## Authors' contributions

PFC analyzed BAC-end sequences and designed primers for BSSRs, evaluated B and GSSRs in 3 mapping populations, mapped the polymorphic SSRs onto the carrot reference map, evaluated part of the markers performance across Apiaceae taxa, interpreted data and wrote most sections of the manuscript. SMC analyzed hybridization-based derived sequences and designed primers for GSSRs, evaluated GSSRs in one mapping population and across Apiaceae species and wrote some sections of the manuscript. SM performed all the experiments and data analyses concerning SSR diversity in the *D. carota *collection and wrote this section of the manuscript. MY, AA and MSA evaluated all the SSRs in the 10117, (PingDing × YC7262) × 8542, and Biennial5 × Criolla INTA mapping populations, respectively, and discussed most sections of the manuscript. MI generated and analyzed EST sequence data, and searched and analyzed EST-derived microsatellites. DAS assisted with all the bioinformatics analysis, and wrote some sections of the manuscript. PWS developed plant populations, initiated design of experiments, discussed and interpreted the data and wrote and revised several sections of the manuscript. All authors revised critically and approved the final manuscript.

## Supplementary Material

Additional File 1 **Table S1 - Characteristics of 300 carrot simple sequence repeat markers**. SSR type, motif, size, sequence, and primers of 300 GSSRs and BSSRs. **Table S2 - Performance of GSSRs and BSSRs in subsets of 7 carrot F_2 _mapping populations**. SSR alleles, polymorphism index, and PCR success_2 _for seven carrot populations. **Table S3 - Accessions used for evaluating carrot SSR marker transferability to other Apiaceae**. A list of the 23 diverse *Daucus *and other Apiaceae evaluated to assess transferability of carrot SSRs to related species. **Table S4 - Potential transferability of SSR markers across Apiaceae accessions**. Amplification success, clarity, and size of PCR products for 300 carrot SSRs when evaluated for 23 diverse Apiaceae. **Table S5 - *Daucus carota *accessions used for analysis of SSR genetic diversity**. A list of the 71 diverse *Daucus carota *evaluated to assess SSR diversity. Click here for file

Additional File 2 **Program used for finding ORFs in sequences**. Click here for file
